# Mitochondrial genome of *Bolanusoides shaanxiensis* (Cicadellidae: Typhlocybinae: Typhlocybini), with its phylogenetic analysis

**DOI:** 10.1080/23802359.2019.1698991

**Published:** 2020-01-10

**Authors:** Bin Yan, Hao-Xi Li, Xiao-Fei Yu, Mao-Fa Yang

**Affiliations:** a College of Agriculture, Guizhou University, Guiyang, China;; b Guizhou Provincial Key Laboratory for Agricultural Pest Management of the Mountainous Region, Institute of Entomology, Guizhou University, Guiyang, China;; c College of Tobacco Science, Guizhou University, Guiyang, China

**Keywords:** Mitogenome, Typhlocybini, *Bolanusoides shaanxiensis*, phylogeny

## Abstract

The mitochondrial genome of one leafhopper species *Bolanusoides shaanxiensis* was sequenced and annotated. The mitogenome is 15,724 bp in length, containing 37 typical genes and a control region. The A + T content of the whole mitogenome is 78.9%. Most of PCGs started with ATN and stopped with TAA, except for *ATP8* started with TTG, *COX2*, *COX3* and *ND5* used incomplete T as stop codon. The phylogeny tree is monophyletic among 31 related species. The relationships of *B*. *shaanxiensis* and *Typhlocyba* sp. were closer than others. This study further enriched mitogenome database of the tribe Typhlocybini.

The genus of *Bolanusoides* was established by Distant (1918), which belonged to the tribe Typhlocybini of the subfamily Typhlocybinae (Hemiptera: Cicadellidae) and included 13 species (Yan 2019). Among of them, two groups were categorized: *B*. *heros* group and* B*. *bohater* group (Dworakowska 1993). The species of *B. shaanxiensis* was described as a new species from China, which is belonged to *B*. *heros* group (Huang and Zhang 2005).

A male adult of *B*. *shaanxiensis* was collected from Dashahe Nature Reserve, Guizhou, China (107°61.4′E, 29°15.8′N), in September 2018. Total DNA was extracted from entire body without its abdomen using the DNeasy Blood and Tissue Kit (Cat. No. 69504). And voucher specimen’s genome DNA and male genitalia were deposited in the Institute of Entomology of Guizhou University, Guiyang, China (GUGC), accession number of them is GUGC-IDT-00188 (Zhang 2018). Then, the mitochondrial genome (mitogenome) of *B*. *shaanxiensis* was sequenced by Illumina NovaSeq6000 platform (Berry Genomics, Beijing, China). The reads were assembled and annotated using Generous Prime (v2019.1.3.). All tRNA genes were identified by ARWEN v1.2 (Laslett and Canbäck 2008). The annotated sequences of mitogenome were submitted to GenBank with accession number MN661136. All protein condoning genes (PCGs) were aligned using MAFFT algorithm in the TranslatorX (Katoh et al. 2017), and then poorly aligned results were removed by Gblocks 9.1 b (Castresana 2000; Abascal et al. 2010). Phylogenetic tree was reconstructed based on the 1st and 2nd codon positions of 13 PCGs using the GTR + I + G model determined by MrBayes3.2.7. on Cipres platform among *B*. *shaanxiensis* and 30 reference species.

Mitogenome of *B*. *shaanxiensis* has 15,724 bp in length, and including 37 typical genes (13 PCGs, 22 tRNA genes and 2 rRNA genes), and a control region. The A + T content of genes are 78.9%, which is similar to other typhlocybine leafhopper mitogenomes (Liu et al. 2017; Song et al. 2017, 2019; Zhou et al. 2016). Most of PCGs used standardized start codon ATN and stop codon TAA, except for *ATP8* started with TTG, and *COX2*, *COX3* and *ND5* genes used incomplete T as stop codon. The length of 22 tRNA is range from 61 bp (*tRNA*-*A*) to 72 bp (*tRNA*-*K* and *tRNA*-*V*). Genes of *16S rRNA* and *12S rRNA* are 1,174 bp and 789 bp, respectively. The results of phylogeny confirmed that relationship of selected taxon categories was monophyletic. The species of *B*. *shaanxiensis* and *Typhlocyba* sp. were clustered into one clade, indicating closest relationship. This work further enriched mitogenome database of the tribe Typhlocybini, with facilitating future studies on Taxonomy and Molecular systematics (Figure 1).

**Figure 1.**
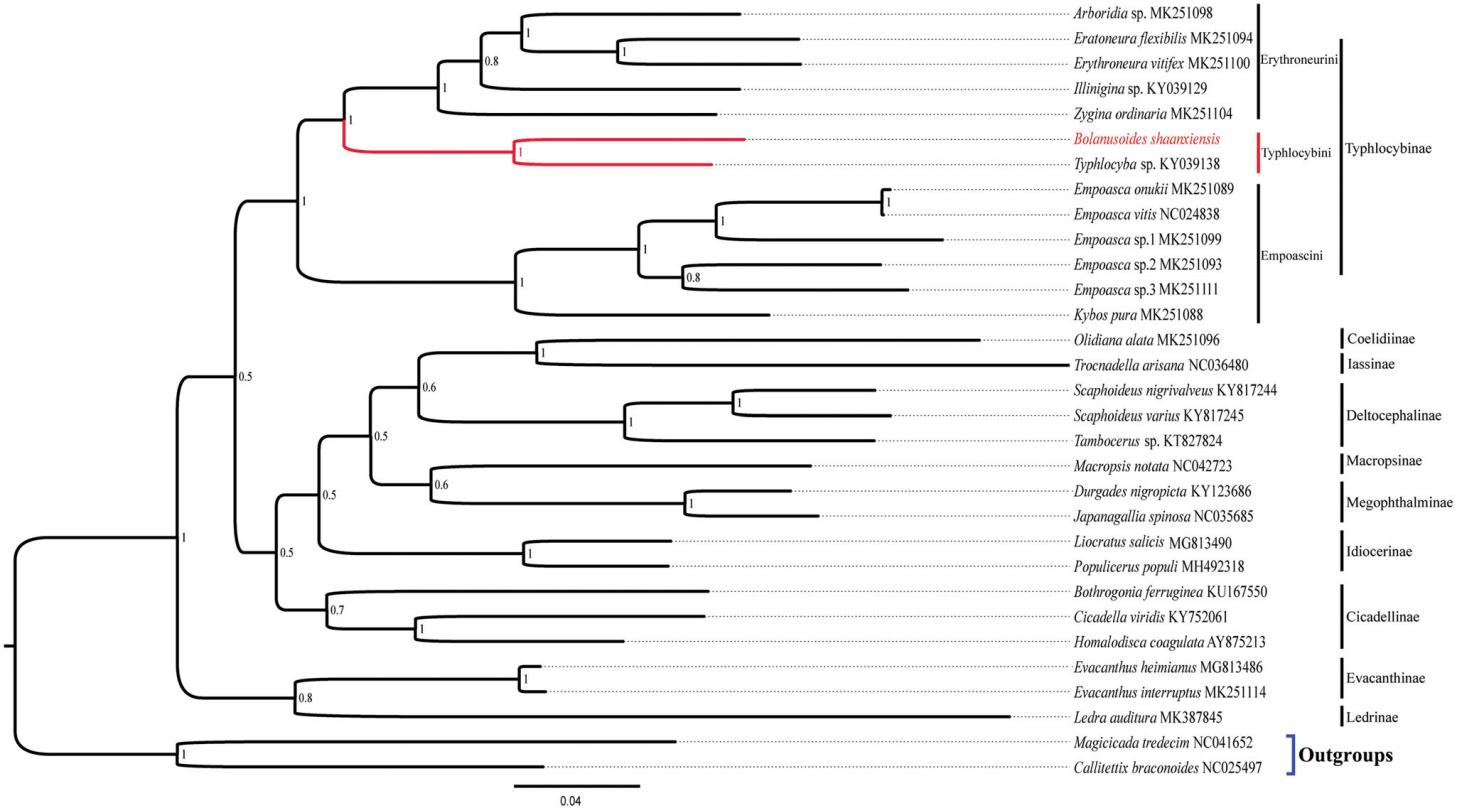
Phylogenetic analysis of *Bolanusoides shaanxiensis* based on the 1st and 2nd codon positions of 13 PCGs. (Numbers at nodes are bootstrap values. The GenBank accession number for each species is indicated after the scientific name.).
